# Metal Nanoparticle-Based Biosensors for the Early Diagnosis of Infectious Diseases Caused by ESKAPE Pathogens in the Fight against the Antimicrobial-Resistance Crisis

**DOI:** 10.3390/bios14070339

**Published:** 2024-07-11

**Authors:** Juan Carlos Gutiérrez-Santana, Viridiana Rosas-Espinosa, Evelin Martinez, Esther Casiano-García, Victor Rafael Coria-Jiménez

**Affiliations:** 1 Laboratorio de Bacteriología Experimental, Instituto Nacional de Pediatría, Insurgentes sur 3700-C, Col. Insurgentes Cuicuilco, Coyoacán C.P. 04530, Mexicovcoriaj@pediatria.gob.mx (V.R.C.-J.); 2 Doctorado en Ciencias Biológicas y de la Salud, Universidad Autónoma Metropolitana, Campus Xochimilco, Calzada del Hueso 1100, Col. Villa Quietud, Coyoacán C.P. 04960, Mexico; 2233800552@alumnos.xoc.uam.mx; 3 Departamento de Sistemas Biológicos, Universidad Autónoma Metropolitana, Campus Xochimilco, Calzada del Hueso 1100, Col. Villa Quietud, Coyoacán C.P. 04960, Mexico; 2192032707@alumnos.xoc.uam.mx

**Keywords:** biosensing techniques, metal nanoparticles, early diagnosis, drug resistance, multiple, bacterial, communicable diseases, infectious diseases, ESKAPE pathogens

## Abstract

The species included in the ESKAPE group (*Enterococcus faecium*, *Staphylococcus aureus*, *Klebsiella pneumoniae*, *Acinetobacter baumannii*, *Pseudomonas aeruginosa* and the genus *Enterobacter*) have a high capacity to develop antimicrobial resistance (AMR), a health problem that is already among the leading causes of death and could kill 10 million people a year by 2050. The generation of new potentially therapeutic molecules has been insufficient to combat the AMR “crisis”, and the World Health Organization (WHO) has stated that it will seek to promote the development of rapid diagnostic strategies. The physicochemical properties of metallic nanoparticles (MNPs) have made it possible to design biosensors capable of identifying low concentrations of ESKAPE bacteria in the short term; other systems identify antimicrobial susceptibility, and some have been designed with dual activity in situ (bacterial detection and antimicrobial activity), which suggests that, in the near future, multifunctional biosensors could exist based on MNPs capable of quickly identifying bacterial pathogens in clinical niches might become commercially available. This review focuses on the use of MNP-based systems for the rapid and accurate identification of clinically important bacterial pathogens, exhibiting the necessity for exhaustive research to achieve these objectives. This review focuses on the use of metal nanoparticle-based systems for the rapid and accurate identification of clinically important bacterial pathogens.

## 1. Introduction

Antimicrobial resistance (AMR) is a phenomenon that has occurred constantly and naturally throughout the evolutionary history of the planet [1,2,3]. It is characterized by the ability of a microorganism to acquire or evade the inhibitory mechanisms exerted by an antimicrobial agent (antiseptics, antibiotics, antifungals, and antiparasitics, among others [1]) to which it was previously susceptible [3,4,5].

The problem has become more evident since the 1950s, when the majority of *Staphylococcus aureus* (*S. aureus*) isolates implicated in hospital and community outbreaks exhibited resistance to penicillin, an antibiotic that is commonly used in eradication therapy for infections caused by this Gram-positive bacterium [3,6]. In the last two decades, the incidence of infections caused by Gram-negative bacteria with AMR has increased rapidly [7]. The identification of multidrug-resistant (MDR), extreme-drug-resistant (XDR) and even pandrug-resistant (PDR) microorganisms [8,9,10] decreases the effectiveness of the available antibiotic therapies [3,11,12], resulting in substantial increases in the morbidity and mortality of infections caused by these pathogens [4,6,13].

The Centres for Disease Control and Prevention (CDC) reported (2013) that approximately two million people annually are infected by AMR bacteria, of whom at least 23,000 die, just in the United States (USA) [4]. In 2017, the global figure approached 700,000 deaths [14], and some reports published two years later included data on disabilities caused by infectious diseases caused by this type of pathogen, which included only the USA and the European Economic Area (approximately 900,000 cases were counted [15]).

By 2050, the estimated number of deaths caused by AMR-related infectious diseases could reach 10 million [14,16,17]; AMR is the leading cause of death in humans after ischaemic heart disease, stroke, diabetes, and cancer [16]. At the end of 2020, the World Health Organization (WHO) reported lower respiratory tract infections, septicaemia, and neonatal infections within the second and third blocks of the main causes of death [18]. Likewise, a study that included 204 countries, carried out prior to the COVID-19 pandemic, generated predictive statistical models that estimated approximately 4.95 million deaths associated with AMR bacteria [18]. These findings suggest that previous estimates could be exceeded if the necessary measures are not taken [17], making it clear that AMR has become one of the greatest public health and environmental problems of 21st century [5,19].

**Figure 1:**
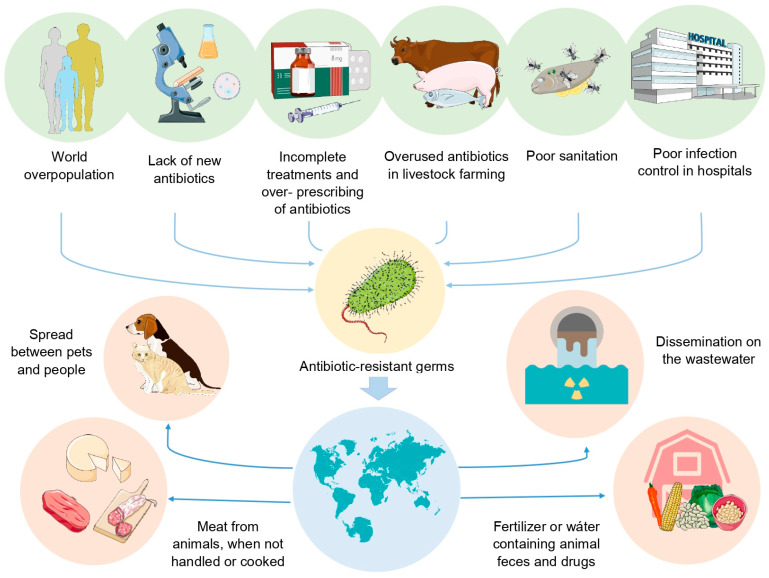
Factors identified by CDC and WHO that have been associated with the emergence of microorganisms with high antimicrobial resistance and their accelerated spread and dispersal of both the pathogen and AMR mechanisms between different microorganisms in different environments, favouring the emergence of increasingly antimicrobial resistant pathogens.

The repercussions of this health problem have led some authors to define it as a global health crisis [20,21,22], which has grown rapidly due to various factors (Figure 1), and the search for rapid diagnostic tools for infectious diseases has been of great interest because an accurate diagnosis is crucial for receiving adequate antibiotic therapy and improving the prognosis of the host [23,24]. In that respect, the unique and diverse properties of nanomaterials (NMs) formulated with metals have attracted attention for a wide range of applications, such as the diagnosis of infectious diseases [25].

The physicochemical properties of metallic NMs have enabled the development of new diagnostic devices or strategies that can function as sensors [26], as well as to be used in combination with existing diagnostic tools, improving the sensitivity or specificity of such equipment [15].

Therefore, the objective of this review is to discuss the current state of the art of early diagnostic approaches based on the use of metallic nanoparticles (MNPs) to develop biosensors that allow for the rapid identification of bacterial pathogens of clinical importance, which is essential in the battle against the AMR crisis.

## 2. ESKAPE Pathogens

Due to the rapid emergence of pathogens with AMR characteristics in hospital settings and their impact on the high incidence of nosocomial infections, some scientific societies, such as the Infectious Diseases Society of America (IDSA), have referred to a set of six opportunistic bacterial species responsible for a high percentage of these infections; these species are referred to as the ESKAPE pathogens [27,28,29,30,31].

ESKAPE refers to the species *Enterococcus faecium* (*E. faecium*), *S. aureus*, *Klebsiella pneumoniae* (*K. pneumoniae*), *Acinetobacter baumannii* (*A. baumannii*), *P. aeruginosa* and *Enterobacter* spp., as well as their high innate capacity to “escape” or evade the lethality of different antimicrobial compounds [3,32,33]. The ubiquitous environmental distribution of these microorganisms favours the acquisition and rapid dispersal of resistance genes (ARGs) [27,33], an aspect that contributes significantly to accentuating the AMR crisis due to the presence of these pathogens in diverse environments, such as water, soil, animals, and humans, which favours the spread of ARGs to different microorganisms [33].

In February 2017, ESKAPE pathogens were a significant part of the list issued by the WHO in response to the AMR crisis, and the main objective of this list was to promote the research and development of new antimicrobial agents [34,35].

**Figure 2:**
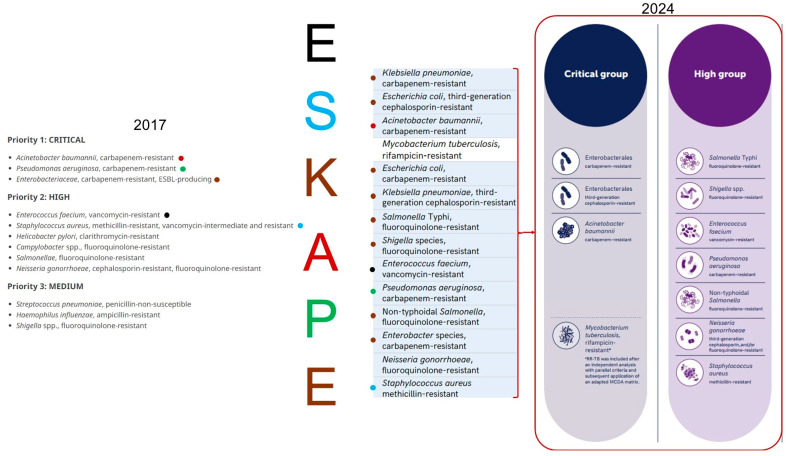
The ‘critical’ category of the list published in 2017 by the WHO consisted of the ‘KAPE’ group, specifically carbapenem-resistant (CR) and extended-spectrum β-lactamase (ESBL)-producing *K. pneumoniae* and *Enterobacter* spp., together with *A. baumannii* and *P. aeruginosa*, both with CR, while the “high” category comprises, among other bacterial species, the “ES” group, in particular vancomycin-resistant *E. faecium* (VRE) and vancomycin- and methicillin-resistant *S. aureus* (VRSA-MRSA). By 2024, the critical group consisted of genus *Enterobacteriaceae* and *A. baumannii* CR, the other species of the ESKAPE pathogens were distributed in the high group. Figures taken and modified from the 2017 and 2024 WHO reports [38,40].

According to the information detailed on the official website of the WHO, the list was scheduled to be updated in 2022 [36] and published in the first quarter of 2023, expanding the objectives towards promoting the development of vaccines and diagnostic tools against the microorganisms included in it [37]. The updated list of priority pathogens was published on 17 May 2024 [38], distinguishing some differences in the distribution of ESKAPE pathogens in the priority groupings of this list (Figure 2), but still are on to the two highest categories (Figure 2) [33,39], demonstrating their importance in global public health [29,35].

## 3. Diagnostic Tools Available for Infectious Diseases

The benefits that a timely diagnosis confers to patients with bacterial infections are widely known [41,42,43,44] and crucially valuable for individuals with diseases caused by microorganisms with a high AMR [23,45,46], as are the bacteria of the ESKAPE group.

Commonly, the microbiological diagnosis of infectious diseases caused by these pathogens is carried out under globally standardized schemes that are supported by the clinical microbiology manuals of different scientific societies, such as the American Society for Microbiology (ASM) or the European Society for Clinical Microbiology and Infectious Diseases (ECCMID), in addition to the guidelines published by different international committees such as the Clinical and Laboratory Standards Institute (CLSI) and the European Committee on Antimicrobial Susceptibility Testing (EUCAST) [27,46,47,48].

These methodologies usually yield complete results (identification of the bacterial species and its antibiogram) after several days (48 h) or even weeks after the acquisition of a biological sample [1,44,49,50]. The effectiveness of the selected antibiotic therapy [51,52], despite being chosen according to the antibiograms [53,54,55], due, among other factors, to the rapid growth of bacteria that, in addition to the different selective pressures to which they are subjected during infection, favours genetic and phenotypic diversification towards a large number of lineages, with antimicrobial susceptibilities that can be different from those identified in the clones isolated and analysed [52]. This is very important for the rapid identification of pathogens in addition to the characterization of their antimicrobial susceptibility.

The use of microbiological cultures, biochemical tests, and microbial visualization by microscopy techniques continues to be valid; these are still considered the starting points for bacterial identification [43,44,56]. However, these methods are not applicable for noncultivable pathogens [27]. Methods based on the identification of antigens by means of antibodies, such as enzyme-linked immunosorbent assays (ELISAs), Western blotting, immunofluorescence (FIA), immunoelectrophoresis and solid phase radioimmunoassays, have been added to these traditional techniques [15,27,57]. In addition, strategies based on nucleic acid analysis or those that use mass spectrometry [27,43,46,58] have led to the development of automated equipment such as MALDI-TOF MS (Bruker Daltonics, Bremen, Germany) [55,59,60], VITEK^®^ 2 (bioMérieux, Marcy I’Etoile, France) [59,61,62], BD Phenix II^®^ (Becton-Dickinson, Rungis, France) [63,64,65] and MicroScan Walk-Away (Beckman Coulter, Inc., Brea, CA, USA) [57,66,67], although the disadvantage of these techniques and these medical devices is that they require prior bacterial culture [27,51].

### 3.1. Nucleic Acid-Based Microbiologic Diagnostic Strategies

Nucleic acid-based strategies include bacterial identification by PCR, real-time PCR (RT–PCR), and quantitative PCR (qPCR) [15,44,51,54]. Multiplex PCR [44,50,51], pulsed field gel electrophoresis [68], and DNA microarrays [15,27,43] are techniques that exceed the sensitivity and specificity of microbiological culture methods [27,44] because they provide results in less time and are able to identify ARGs [27,43,69]. However, these techniques require detectable levels of nucleic acids under conditions of low gene abundance and heteroresistance [27].

Recently, digital PCR has made it possible to identify minimal amounts of pathogens or target molecules without requiring enrichment based on microbiological cultures [15,27]. Unfortunately, this strategy often produces false-negative and false-positive results [27,44]. In addition, the identification of hypervariable microorganisms resistant to carbapenems (CRs) or producers of extended spectrum β-lactamases (ESBLs) acquired by single nucleotide polymorphisms (SNPs) usually does not correlate with resistance phenotypes [27].

The most current molecular techniques available for identifying microorganisms include next-generation sequencing (NGS) and whole-genome sequencing (WGS) [27,43], which allow for the precise identification of microorganisms, bacteria quantification, and the possibility of detecting polymicrobial infections [43]; additionally, in combination with bioinformatic analysis, these techniques accurately identify pathogens and characterize their antimicrobial susceptibility, which is commonly achieved via disk diffusion (Kirby-Bauer), agar diffusion, and minimum inhibitory concentration methods [27,43].

These technologies have made it possible to establish metagenomics (mNGS) as one of the most efficient strategies available for the study of microbial communities, which is practically impossible for microbiological cultures. These techniques avoid the possibility of false-positives or false-negatives, but their detection capacity is limited to the mechanisms described and previously documented information [27].

### 3.2. Microbial Detection Systems Based on Proteomics

Because proteins and their level of expression indicate the functional state of a bacterial cell, proteomic methods have become more relevant in the clinic [27,68]. Initially, proteomic analyses were based on two-dimensional gel electrophoresis (2-DE) and differential gel electrophoresis (DIGE) techniques with fluorescent markers [27]. However, technological advancements in mass spectrometry have made it possible not only to identify proteins but also to quantify them and detect their functional status [27,57]. This process can be achieved by different techniques, such as electrospray ionization (ESI) [27,70] or by means of a laser-assisted desorption/ionization matrix (MALDI) [15,57], while mass analysis can be carried out by means of time-of-flight (TOF) instruments [51,68] or ion mobility spectrometry (IMS) [27]. These techniques have allowed for the accurate identification of microorganisms, although they are limited because they require initial growth in microbiological cultures [51]. A prior spectral library that allows comparison of the “mass fingerprints” obtained from a sample with bacterial reference spectra is needed [27,59,68].

In recent years, the adjustment between the frequency of incident light and its scattering, known as Raman spectroscopy, has made it possible to visualize specific molecular vibrations based on proteins, lipids and DNA that constitute the “chemical fingerprint” of a single bacterial cell (SCRS), which is useful not only in rapid bacterial identification but also in the characterization of antimicrobial susceptibility in approximately four hours [15,27]. Although this technique also requires reference spectra [27], its detection capacity has made this type of spectroscopy one of the most commonly used methods in the development of biosensors [15,51].

## 4. Emerging Technologies for the Diagnosis of Infectious Diseases

The diagnostic strategies that have been used experimentally and in the clinical field are diverse and functional in regard to the identification of bacterial pathogens in most situations [43,51]. This can benefit the health and survival of individuals with infectious diseases [71].

However, all available strategies have deficiencies in specific cases, such as the need for growth in microbial cultures or the need for a library of prior information [51]. This has resulted in the misidentification of some subpopulations of *Klebsiella aerogenes* (*K. aerogenes*), *Mycobacterium tuberculosis* (*M. tuberculosis*), *Burkholderia cepacia* complex (*Bcc*), and *P. aeruginosa*, among others [3,13,44,72,73].

Therefore, different technologies have been explored to develop diagnostic tools capable of identifying and characterizing bacterial pathogens with sensitivities, specificities, precisions, and speeds greater than those currently available [51]. The development of novel techniques, such as typing based on short, grouped, and regularly spaced palindromic repeats (CRISPR), combined with other technologies such as nanotechnology [51,74], has provided relevant information for distinguishing bacterial pathogens [74], with the latter establishing itself as one of the areas of greatest interest for the development of tools aimed at preventing various infectious diseases (such as those focused on the identification of pathogens in food and beverages for human consumption), as well as for their treatment and diagnosis [75].

### Nanotechnology Based on MNPs for Disease Diagnosis

The scientific and technological discipline that uses materials at nanometric scales (NMs) with at least one dimension less than 100 nm is known as nanotechnology [22,76]. This field has grown rapidly in recent decades [22,75] and has given rise to other disciplines, such as bionanotechnology, focused on the development of nanoscale materials and devices with unique properties resulting from the combination of the qualities of NMs with different biomolecules, as well as the use of the properties of these systems. In biology [77], nanomedicine refers to the use of these technologies exclusively in the medical field [22,78].

Among the different NMs [76], those formulated from metals (MNPs) have shown interesting physicochemical properties in regard to their application in nanomedicine [22], among which a high surface area ratio, mechanical resistance, and optical, electronic, magnetic, chemical, spectral, and plasmonic properties, among others, stand out [22,75,76,79]. However, the parent metal is not commonly present [76].

MNPs can be easily modified [80]. In addition, their properties vary depending on their size, shape, and degree of dispersion [76]. In addition to their coupling with different biomolecules or functional groups [76,79,81], small molecular ligands, such as amphiphilic surfactants, polymers, peptides, nucleic acids, and aptamers [81,82,83,84,85], allow MNPs to have additional functions [76,81] and increase their sensitivity and specificity for a specific biomarker [79,83].

In nanomedicine, the most commonly used MNPs are those of iron oxide [86,87,88,89], such as magnetite (Fe_3_O_4_) [79,90,91] or its oxidized form maghemite (Fe_2_O_3_) [79,90,92], combined with those synthesized from nickel (NiNPs), cobalt (CoNPs) [90,93], and copper (CuNPs) [79,94,95]; however, the optical, spectral and plasmonic properties of MNPs based on noble metals, such as gold (AuNPs) or silver (AgNPs), have made them the most studied for their application in bioimaging [75,76], diagnosis, therapy, and research [79,81].

Specifically, in the diagnosis of diseases, systems based on MNPs have demonstrated their potential utility for rapid and efficient diagnosis, as well as for the monitoring of diseases [79]. Moreover, some configurations can exhibit dual activity, allowing them to function as diagnostic and therapeutic agents [79,95,96].

Advances in the identification of important biomarkers in different diseases, such as the overexpression of the transmembrane protein tyrosine kinase-7 (PTK7) in T cells in acute lymphoblastic leukaemia [97,98,99], the platelet-derived growth factor (PDGF) in the diagnosis of different types of tumours [100], and the proteins fetuin A and fetuin B present at low concentrations in the blood of individuals with Alzheimer’s disease (in contrast to high levels of clustering [101]), among many other markers and diseases, have allowed for the design of diagnostic strategies based on MNPs [97,102].

These biomarkers could be identified with high sensitivities and specificities by combination of MNPs with current commercial equipment, such as flow cytometers [102], different types of microscopes [103,104,105,106], and different types of spectroscopes [22,107,108,109], such as those focused on enhanced surface Raman scattering (SERS), which are commonly used in the diagnosis area and can even identify a specific biomarker with the naked eye, such as lateral flow biosensors (LFBs) [15,25,51,110,111].

## 5. MNPs-Based Biosensors for Pathogen Identification

The International Union of Pure and Applied Chemistry (IUPAC) has defined biosensors as “autonomous devices that are constituted by a receptor (which is a biological element such as tissues, cells, microorganisms, antibodies, enzymes, nucleic acids, among others” [26,112,113]. Some authors have defined bioreceptors [112,114,115]) and physicochemical transducers (encompassing electrochemical, optical, calorimetric, thermometric, or mass-based strategies [79,112,113]), which translate biological signals into a measurable signal [116] and must be proportional to the concentration of a specific analyte or biomarker [26,82,115].

Biosensors must show high specificity towards a specific biomarker [26,113] and function adequately even in complex biological samples and under diverse conditions in terms of pH, temperature, and other physical parameters [26]. This has allowed for the development of biosensors for the rapid identification of infectious diseases of global importance, such as diseases caused by severe acute respiratory syndrome coronavirus 2 (SARS-CoV-2) [26,117,118], hepatitis [119], and gonorrhoea [120], as well as those caused by different bacterial pathogens [115].

The incorporation of NMs in the development of biosensors has opened new opportunities for the detection of biomarkers at minute concentrations, such as attograms [112]; the design of biosensors with greater specificity, sensitivity [26,115], and reproducibility [113,115]; and the possibility of developing compact and portable devices, which some authors have called “nanobiosensors” [26,79,112,115], allowing for greater accessibility and ease of use [26,112,113].

Properties such as surface plasmon resonance (SPR) [113,121,122]; the metal-enhanced fluorescence effect (MEF) [83,102]; and the optical, electrical, magnetic and photothermal qualities [94,96,104,123] of MNPs have allowed for the development of different types of sensors focused on the early detection of the causative agents of different infectious diseases [26,80,124].

Some examples of this include the work by Yang H. et al. (2014), who used magnetic NPs (MagNPs) of Fe_3_O_4_ to develop a biosensor for the early detection of hepatitis B virus (HBV). The results showed high intensities of chemiluminescence (CL) for HBV in contrast to low intensities for other viruses, such as hepatitis C (HCV) and acquired immunodeficiency virus (HIV), as well as *Escherichia coli*. The method consisted of a previous PCR amplification of a specific genetic region of HBV using a biotinylated nucleotide (2′-deoxyuridine, 5′-triphosphate (dUTP) to obtain biotinylated amplicons, which were captured by hybridization between biotin and carboxymethylated glycans (CMG) immobilized on MagNPs. CL was detected with the addition of streptavidin-modified alkaline phosphatase (SA-AP), followed by 3-(2′-spiroadamantane)-4-methoxy-4-(3′-phosphoryloxy)-phenyl-1,2-dioxetane (AMPPD), achieving limits of detection (LODs) of 0.5 pM in less than 1 h [119].

Chen F. et al. (2020) took advantage of the light diffraction properties of AuNPs to identify *Chlamydia pneumoniae* (*C. pneumoniae*) by dark field microscopy. The detection strategy started by coupling an anti-*C. pneumoniae* antibody to a 26 nucleotide (n) single chain DNA sequence (n) complementary to a ssDNA sequence immobilized on 15 nm AuNPs. Thus, through antigen–antibody interactions, hundreds of complexes surrounded this Gram-negative coccus, resulting in the formation of a crown-shaped structure due to strong light scattering and allowing LODs of 4 CFU·µL^−1^ in <30 min [103].

For SARS-CoV-2, efficient biosensors have been developed for the early detection of the virus [117,125]. For example, Gao Y. et al. (2021) chemically synthesized AuNPs of ~17 nm, on which four cyanine 3 (Cy3)-labelled ssDNA probes were absorbed and proved complementary to the viral RNA sequence of the open reading frame (ORF1ab) and the genes that encode the viral envelope (E). Under this configuration, the biosensor functions in three ways, as described below:

The ssDNA probes bind by complementarity with the specific regions of the viral RNA, resulting in a decrease in the dispersion intensity detected by SERS in a manner dependent on the RNA concentration of SARS-CoV-2.

The identification led to the weakening of the absorption peak at an optical density (OD) of ~520 nm, complemented by the increase in the absorbance peak at an OD of 690 nm (OD_690_).

Those spectral behaviours were the result of a greater aggregation of the AuNPs due to the formation of ssDNA-RNA complexes, which are identified by the naked eye by the change in colour of the solution (blue to red), identified by the increase in fluorescence at OD_570_.

This biosensor exhibited the capacity to recognize single-base mismatch in each working mode, minimizing the false negative/positive reading of SARS-CoV-2 with LODs of 160 fM, 259 fM and 395 fM by UV/Vis spectrophotometry, fluorescence and SERS, respectively, in ~40 min [117].

In 2023, Dighe K. et al. developed a biosensor consisting of ssDNA sequences complementary to specific sequences of the cryptic plasmid DNA (ORF6) of *Chlamydia trachomatis* (*C. trachomatis*) and to the sequence that encodes the outer membrane protein (NGK_2093) from *Neisseria gonorrhoeae* (*N. gonorrhoeae*). The ssDNAs were coupled to AuNPs and agglomerated by specifically recognizing both pathogens without reacting with *Staphylococcus aureus* (*S. aureus*), *Acinetobacter baumannii* (*A. baumannii*), *Escherichia coli* (*E. coli*), *Bacillus subtilis* (*B. subtilis*), or *Streptococcus mutans* (*S. mutans*). Its evaluation in 60 clinical samples of cervical smears and urine samples showed a sensitivity of ~100%, with LODs of 5 copies/µL for *N. gonorrhoeae* and 5 copies/µL for *C. trachomatis* [120].

Despite the different properties of MNPs that are potentially useful for early and accurate diagnosis of the causative agents of infectious diseases, efforts have commonly focused on exploiting the antimicrobial qualities of MNPs for the design of therapeutic tools, as evidenced by the identification of 1706 results derived from a search in PubMed and 451 in the Web of Science carried out on 1 March 2024 using the MeSH terms therapy, treatment, human infections, infectious diseases, communicable diseases, and metal nanoparticles in combination with the Boolean operators AND and OR as follows: ((metal nanoparticles) AND ((therapy) OR (treatment))) AND (((human infections) OR (infectious diseases) OR (communicable diseases))). For early diagnostic approaches, only 179 and 54 results were obtained in PubMed and Web of Science, respectively, using the following search structure: ((metal nanoparticles) AND (early diagnosis)) AND ((human infections) OR (infectious diseases) OR (communicable diseases))). The results were further reduced by focusing exclusively on the ESKAPE pathogens, without obtaining results for *E. faecium*, *K. pneumoniae* and *Enterobacter* spp., and there were only six articles for *S. aureus*, two for *P. aeruginosa*, and one for *A. baumannii* in PubMed, in contrast to only two results for *S. aureus* in Web of Science. The following paragraphs describe the strategies used for the early identification of ESKAPE pathogens based on biosensors developed with MNPs.

### 5.1. MNP-Based Nanobiosensors for the Identification of Multiple ESKAPE Species

There are several systems designed to identify different pathogenic bacteria, one of which was designed by Chan PH. et al. (2013), who encapsulated gold nanoclusters (AuNCs) in lysozymes (lysozyme-AuNCs) with the prior knowledge that the latter can recognize and bind to peptidoglycans of the bacterial cell wall. Under this principle, the addition of the lysozyme−AuNC complex to a bacterial sample solution allowed for the formation of lysozyme−AuNC−bacteria conjugates, which were easily concentrated by centrifugation and visible to the naked eye as a solution with red emission when exposed to ultraviolet light. The identification of the bacteria recovered from the samples was carried out with automated MALDI-MS equipment, which achieved LODs of ~10^6^ colony forming units (CFU)·mL^−1^ in assays with a duration of ~1 h, demonstrating the ability to identify *E. coli*, *K. pneumoniae*, and *P. aeruginosa*, as well as PDR variants of *A. baumannii*, *S. aureus*, and *Enterococcus faecalis* and vancomycin-resistant variants of the latter (VRE) [126].

Subsequently, researchers headed by EI Ichi S. (2014) developed a conductometric biosensor for the rapid and highly sensitive detection of Gram-negative bacteria. The biosensor consisted of the capture of microorganisms based on antibodies against lipopolysaccharides (anti-LPS) specific for different bacterial species that were previously conjugated with carboxylated superparamagnetic particles and fixed by magnetism to the electrodes of a conductometric transducer. Thus, when these modified electrodes were introduced into solutions inoculated with bacteria, a decrease in the conductance of the electrode was observed due to the capture of microorganisms on its surface, exhibiting LODs ranging from 1 CFU·mL^−1^ for *E. coli* and *Serratia marcescens* (*S. marcescens*) and from 10 to 10^3^ CFU·mL^−1^ for *P. aeruginosa* and *A. baumannii* in just 2 min; additionally, this technique allowed for the detection of bacterial concentrations that were undetectable by traditional immunoblot techniques, and Gram-positive bacteria did not significantly change the sensor impedance [127].

**Figure 3:**
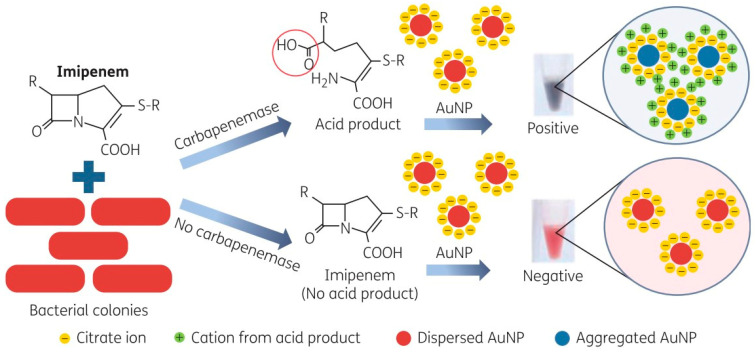
Methodologic principle of the GoldC test described by Srisrattakarn A. and colleagues [128], which is based on hydrolysis of the β-lactam ring of imipenem by carbapenemases, resulting in an acid product. In the presence of acid, the repulsive force between the AuNPs is eliminated, and thus the inter-particle distance is decreased. This leads to AuNP aggregation, which can be detected by the change in the AuNP solution from red to purple, blue, or green (positive). The AuNP solution remains red (negative) in the absence of acid production. Scheme taken from Srisrattakarn A. et al. [128].

Later, a group of researchers led by Srisrattakarn A. (2017) developed a colorimetric biosensor to detect the production of carbapenemases. The sensor consisted of AuNPs of ~12 nm obtained by citrate reduction (GoldC), which resulted in solutions with an intense red colour due to the presence of monodisperse particles. The functional principle of this system was based on the identification of the hydrolysis of the β-lactam ring of imipenem (IMP) by the action of carbapenemases, resulting in the acidification of the solution that decreased the repulsive force between the AuNPs, favouring their grouping, which was visualized as a colour change towards violet, blue, or green solutions for carbapenemase-producing bacteria and no colour change (intense red colour) for bacteria that do not produce these enzymes (Figure 3). The system was compared against commonly used tests such as Carba NP (CNP) and the specific test for *A. baumannii* (CarbAcineto NP; CAcNP) in a total of 99 clinical isolates of *A. baumannii*, *Pseudomonas* spp. and carbapenemase-producing *Enterobacteriaceae* (CPE), as well as in 89 non-carbapenemase-producing variants (non-CPE), demonstrating a sensitivity of 100% in the identification of *Pseudomonas* spp. and CPE (for the traditional CNP test, the sensitivity was 98.6%). Similarly, GoldC exhibited a sensitivity of 96.7% for recognizing isolates of *A. baumannii* producing carbapenemases, while the sensitivity of CAcNPs was 93.3% in assays with a duration of only 5 min [128]. This system shows that it is possible to design devices or platforms capable of quickly identifying pathogenic bacteria and identifying their susceptibility to different families of antimicrobial agents. The implementation of this technique in the clinic could accelerate therapeutic decision-making, which in turn could promote therapeutic success and thus a better prognosis.

In 2018, Tominaga T. developed an immunoassay under the LFB principle for the rapid detection of two genera of opportunistic bacteria (*Klebsiella* and *Raoultella* of the *Enterobacteriaceae* family). Briefly, the author immobilized anti-*Klebsiella* monoclonal antibodies (pAbs) on a nitrocellulose membrane (NC), and, in parallel, the same antibodies were labelled with palladium NPs (PdNPs). The operation of the system consisted of adding the PdNPs coupled to pAbs to capture the bacteria, followed by taking an aliquot of that mixture to deposit on the LFB, where the PdNP−pAb−bacteria conjugates migrated by capillary action along the NC membrane until they were captured by the antibody. Antibody capture was visualized in ~15 min as a red line due to the colloidal aggregation of the metal, which was complemented with the identification of the urease activity by means of the addition of 1% urea solution, resulting in an increase in pH ≥ 1 for bacteria that express said enzyme. This LFB was evaluated in 72 bacterial strains, and the results showed that the device was able to identify the strains corresponding to *K. pneumoniae*, *Klebsiella oxytoca* (*K. oxytoca*), and *Raoultella ornithinolytica* (*R. ornithinolytica*), as well as differentiate the bacteria of the genus *Klebsiella* by urease activity in ~3 h. Likewise, when analysing food samples, the device showed an accuracy of 73% (19/26) in the detection of bacteria of the *Klebsiella* group. One of the issues to overcome is the fact that there are strains of this genus that do not produce urease [129], which explains the low percentage of positivity due to false negatives.

The following year, Lee C. et al. (2019) designed an amperometric biosensor on a silicon chip covered with polydimethylsiloxane (PDMS), on which silicon dioxide (SiO_2_) nanochannels were traced that interconnected a loading region (where the authors deposited ~20 µL of a blood plasma sample inoculated with *P. aeruginosa* and *S. aureus*) with a loading region of redox-active AuNPs (raAuNPs) coupled to species-specific monoclonal antibodies, specifically in a region delimited with multiple-walled carbon nanotube filters (MWCNTs), which they called the “incubation chamber”, a section in which the raAuNP–bacteria conjugates were retained and facilitated the elimination of unconjugated raAuNPs. The complexes were separated from the MWCNTs by passing 5–10 mL of phosphate saline buffer (PBS) in the opposite direction to the initial flow, and this solution was deposited in the “measurement chamber”, which was connected to a bipotential electrochemical workstation. Due to the high electrical conductivity of the raAuNPs, both pathogens were identified with LODs of 10 CFU mL^−1^ in just 30 min [130].

In 2022, Xie G. et al. synthesized MagNPs with photothermal qualities (Fe_3_O_4_@C) and incorporated them into hydrogels composed of hydroxyethyl methacrylate (HEMA) and acrylamide (AAm) with the aim of developing a hydrogel photothermal photonic (HPP), which allowed for the identification of bacterial infections with the naked eye by the colour of HPP changing towards blue tones due to the reduction in pH of the infected site caused by the bacterial metabolism of glucose. In addition, in vitro and in vivo tests performed on pig skin wound models inoculated with *E. coli* and *S. aureus* demonstrated that the device has the ability to disinfect wounds through light irradiation in regions close to the infrared (NIR) region for 15 min, eliminating 100% of both bacterial species [131], suggesting the possibility of developing dual systems with the ability to detect bacterial pathogens in vivo and in situ, as well as to exert antimicrobial activity on them.

Later, Wen CY. et al. (2023) developed a colorimetric sensor for the multiple and simultaneous detection of *S. aureus*, *S. typhimurium* and SARS-CoV-2 in ~40 min through the combined use of MNPs coupled to specific antibodies for these species (anti-*S. aureus*, anti-*S. typhimurium* and anti-spike) as follows: AgNPs conjugated with anti-*S. aureus* formed a “yellow immuno-reporter” for *S. aureus* (IY-SA), AuTNPs conjugated with anti-*S. typhimurium* formed a “blue immuno-reporter” for *S. typhimurium* (IB-ST), and AuNPs coupled with anti-spike formed a “red immuno-reporter” for SARS-CoV-2, whose combination in a single solution resulted in a black liquid. However, the presence of one or more of the aforementioned pathogens, followed by their separation by magnetism, resulted in colour changes in the solution that were detectable by the naked eye, which, together with their spectral analysis, made it possible to distinguish LODs of 10 CFU·mL^−1^ for *S. aureus* and *S. typhimurium* and 0.2 μg·mL^−1^ for the spike protein [132].

Li, J. et al. (2023) developed an LFB for the rapid and early detection of *S. aureus* and *Streptococcus pneumoniae* (*S. pneumoniae*) in respiratory infections. For this purpose, the authors used strips consisting of an absorbent pad, a pad for loading the sample, and an NC membrane on which anti-*S. aureus* and anti-*S. pneumoniae* antibodies were immobilized (test line 1 and test line 2, respectively) to visualize and identify the bacterial capture of both microorganisms through the use of MagNPs of Fe_3_O_4_ covered with polyethyleneimine (PEI), on which they immobilized AuNPs (Fe_3_O_4_@Au).

This nanocomposite was treated with 5,5-dithiobis-(2-nitrobenzoic) (DTNB), which is an active molecule for detection by SERS (Fe_3_O_4_@Au/DTNB) and on which they coupled colloidal AuNPs (Fe_3_O_4_@Au/DTNB/Au) coated with 4-mercaptophenylboronic acid (4-MPBA), a compound that has shown affinity for bacterial peptidoglycans, lipopolysaccharides, and glycoproteins [133,134], establishing the “Fe_3_O_4_@Au/DTNB/Au/4-MPBA” complex.

The described method consisted of adding the Fe_3_O_4_@Au/DTNB/Au/4-MPBA complex to solutions and sputum samples inoculated with these bacteria, allowing their capture by the action of 4-MPBA and concentration through magnetic separation. Due to the nucleus of the nanocomposite (MagNPs of Fe_3_O_4_), the resulting bacterial solution was subsequently loaded into the LFB, allowing the identification of microorganisms through their capture by specific antibodies (anti-*S. aureus* and anti-*S. pneumoniae*) by means of the appearance of bands; analysis by SERS showed LODs of 8 and 13 CFU·mL^−1^, respectively, in ~20 min [135].

Recently, Huang X. et al. (2023) treated AgNPs with chloroauric acid (HAuCl_4_) and chloroplatinic acid (H_2_PtCl_6_) to form “UAA@P NPs”, on which they immobilized 4-MPBA for bacterial capture, calling this complex “UAA@P/M NPs”. These nanoparticles were added to blood samples inoculated with *S. aureus*, favouring the formation of UAA@P/M NP–bacteria complexes, followed by their loading in lateral flow strips. LFTSs previously prepared with specific antibodies for the species immobilized on the NC membrane were able to selectively capture the UAA@P/M NP–bacteria complexes, which were identified by means of a colorimetric method (LOD 1 × 10 ^3^ CFU·mL^−1^), SERS (LOD 3 CFU·mL^−1^), photothermal (LOD 27 CFU·mL^−1^), and a catalytic approach (LOD 18 CFU·mL^−1^). Thus, by adding selective antibodies for *E. coli* and *S. aureus* to the device, as well as its application for the analysis of clinical blood samples, the authors demonstrated the ability of this device to distinguish between infected patients and healthy individuals as well as to differentiate three types of bacterial pathogens (*S. aureus*, *E. coli* and *P. aeruginosa*) [133].

### 5.2. MNP-Based Biosensors Focused on S. aureus

Wang, J. et al. (2017) developed a system for the enrichment and identification of pathogens based on MagNPs of Fe_3_O_4_ coupled to chlorine e6 (Ce6), a compound that is commonly used as a photosensitizer, and the immobilization of selective aptamers for *S. aureus* (Fe_3_O_4_-Ce6-Apt). The procedure consisted of the inoculation of bacteria in blood samples of healthy mice, followed by the addition of Fe_3_O_4_-Ce6-Apt and its concentration by magnetism, followed by staining with SYTO9 and visualization by fluorescence microscopy, achieving an LOD of 10 CFU. These data were confirmed by the infection of healthy mice with the pathogen, followed by incubation for 1 h and the collection of blood samples from the animals to apply the methodology described above, the findings of which were consistent with the results obtained by blood culture (“gold standard”). In addition, the treatment of blood samples previously inoculated with *S. aureus* with NIR for 5 min resulted in total disinfection of the sample, as evidenced by the transfusion of the disinfected sample to healthy mice, which did not cause adverse reactions; this finding can be extrapolated to other species, as demonstrated by the same authors with *E. coli* [136].

Similarly, in 2020, researchers led by Gao X. developed a chip based on a slide covered with a plasmonic gold film and self-assembled monolayers (SAMs) of 4-MPBA, forming the “MPBA/pAu” complex, which detected and captured *S. aureus* by binding 4-MPBA to bacterial peptidoglycan with an LOD < 10^2^ CFU·mL^−1^; the reaction sensitized the cell wall structure, which, through photothermal treatment with NIR, allowed an increase in the temperature of the chip surface (~65 °C) that resulted in bacterial death. The authors performed in vivo tests on wounds of mice inoculated with *S. aureus* and subsequently treated with the chip, which was placed on the wound. The animals were irradiated with NIR for 10 min every 24 h, resulting in a 28% reduction in the wound size at 5 days postinfection coupled with adequate tissue regeneration. Additionally, under this methodological principle, they captured, detected, and eliminated *E. coli* [134].

In the same year, Potluri P. et al. (2020) combined SERS and PCR technology to develop a method for the identification of methicillin-resistant *S. aureus* (MRSA), a strategy based on the use of SERS reporter molecules (4-MPBA and 4-mercapto-3-nitro-benzoic acid [MNBA]) and complementarity-mediated oligonucleotide capture probes, which were coupled to AuNPs. Thus, the researchers used specific primer pairs for the MRSA-characteristic genes *mecA* and *femA*; the anti-sense primer of both genes was labelled with biotin, allowing for the concentration of the amplicons after the addition of streptavidin-modified magnetic beads, followed by magnetic separation and analysis by SERS, as well as the ability to distinguish the specific spectra of both amplicons in the genomic DNA of clinical isolates, exhibiting an LOD of 10^4^ DNA copies in ~80 min [137].

In the same year, Feng, Y. et al. developed an electrochemical biosensor for the detection of *S. aureus* through the formation of SAMs with a DNA hairpin structure (H_1_) on an interdigital gold electrode. In the presence of the specific hypervariable region of the 16S rRNA gene sequence of this pathogen, the stem–loop structure was recognized by a complementary hairpin (H_2_) coupled to AuNPs, allowing for the formation of AuNPs linked to a long product resulting from hybridization between the two hairpins, a process known as hybridization chain reaction (HCR). By adding silver solution to the electrode, the formation of silver threads along the HCR product was favoured, increasing the conductivity of the electrode and yielding an LOD of 50 CFU mL^−1^ in ~100 min [138].

Wang, XY et al. (2020) took advantage of the specificity for *S. aureus* exhibited by the phage M13 heptapeptide, which they used as a substrate for the synthesis of AuNPs, followed by modification with acid 5.5-dithiobis-(2-nitrobenzoic) (DNTB), which acted as an active molecule for SERS. This complex was tested in commercial beverages inoculated with bacteria, and the authors detected the characteristic Raman spectrum of *S. aureus* with LODs as low as 10 CFU·mL^−1^ without interacting with other bacterial species. Additionally, this nanocomposite demonstrated remarkable antimicrobial activity after the first hour of incubation, which was visualized by electron microscopy as bacterial fragmentation, and after 8 h of interaction with the complex, a significant reduction in the viable counts of the pathogen was detected [139].

Using a different approach, Mohamed S. et al. (2020) obtained antibodies specific to a highly conserved polypeptide of the *S. aureus* cell wall and immobilized them on AuNPs. These complexes were added to a specific test line in NC membrane strips, and nonspecific IgG antibodies were fixed in another section of that membrane and used as a control for this LFB. Thus, in loading solutions inoculated with the bacteria, or in loading aliquots of blood samples from neonates infected with *S. aureus*, the antipolypeptide specifically captured the microorganism in just 15 min, with an LOD of 10^2^ CFU mL^−1^, and this interaction was visualized withthe appearance of a reddish band on the LFB [140].

In 2022, Huang, X. et al. developed a sandwich system for the rapid detection of *S. aureus*. The system consisted of AuNPs coupled to a reporter for SERS (4-nitrothiophenol; 4-NTP) and to a poly-A DNA sequence, followed by treatment with HAuCl_4_ and hydroxylammonium chloride (NH_2_OH·HCl) to form bridge AuNPs with nanospaces (AuNNPs), on which they immobilized an aptamer specific for these species (apt-AuNNPs). On the other hand, they used MagNPs of Fe_3_O_4_ covered with SiO_2_ (Fe_3_O_4_@SiO_2_NPs) conjugated with concanavalin A (ConA) (ConA-Fe_3_O_4_@SiO_2_ NPs), which can bind with different residues present in polysaccharides [119,141]. In this way, the system allowed for the specific identification of this Gram-positive coccus with apt-AuNNPs, while the binding of ConA-Fe_3_O_4_@SiO_2_ NPs with the polysaccharides of *S. aureus* allowed for the enrichment of the detectable signals. Magnetic separation via SERS and plasma-coupled mass spectrometry (ICP-MS) achieved an LOD of 11 CFU·mL^−1^ in ~30 min in serum samples inoculated with *S. aureus* in addition to causing bacterial death due to the photothermal properties of the Fe_3_O_4_ MagNPs activated by NIR for 5 min [142].

In parallel, Yi, Y. et al. (2022) designed a MRSA identification system based on a specific aptamer for variants of this pathogen hybridized by complementarity with two short DNA sequences coupled with 20 nm AuNPs coupled to a DNA hairpin and 30 nm AuNPs coupled to 4-NTP covered by a protective DNA sequence to prevent clustering. These three general elements were shown to be activated by the presence of MRSA, which was recognized first by the aptamer, causing the release of the two sequences with which it previously hybridized and subsequently joined by complementarity to a fragment of the DNA hairpin, establishing a double helix section in the apical region of the 20 nm AuNPs, which, after the addition of Exo III exonuclease, was hydrolysed. A small DNA strand was left that, by complementarity, joined the protective DNA of the 30 nm AuNPs, forming a new double-stranded section that was also degraded by Exo III, depriving these AuNPs and favouring their grouping, an effect that increased with the addition of Mg^2+^ buffer. Due to the 4-NTP labelling and the use of a portable Raman spectroscope, it was possible to identify MRSA in ~40 min with an LOD of 1 CFU mL^−1^ [143].

In 2023, a group of researchers led by Li L. created a system consisting of Fe_3_O_4_ MagNPs stabilized with 2-bromo-2-methylpropionic acid (BMPA) (MagNPs@MPA) to cancel their magnetism. They were conjugated with a specific recognition peptide for *S. aureus* (P) and with a “paramagnetic enhancer peptide” (P1) composed of a peptide with metallopeptidase binding motif 2 (MMP-2) (PLGVRG) linked to the KLVFF sequence peptide, designed to form dipeptides between similar peptide sequences (KLVFF motifs) through bonds between phenylalanines (FF). Additionally, gadolinium ions (Gd^3+^) chelated by tetraazacyclododecane tetraacetic acid (DOTA) were linked by hydrogen bonds on this peptide (KLVFF), forming the modified magnetic resonance system with peptides (MRET). In the absence of the enzyme MMP-2 (commonly overexpressed in microenvironments infected by *S. aureus*), these nanocomposites were held together through the FF bonds of the KLVFF motif; however, in the presence of MMP-2, the nanocomposite was disassembled into monomers of MagNPs and Gd^3+^ bound to *S. aureus* through the P peptide, observing its dispersion by transmission electron microscopy (TEM). Additionally, by means of characteristic signals by magnetic resonance imaging (MRI), it was possible to identify in vivo the site of myositis caused by *S. aureus* in infected mice, with an LOD < 10^4^ CFU [144].

Wang C. et al. (2023) used two nanostructures to develop a SERS-based bacterial sandwich biosensor. One of the structures, i.e., the “signal module”, was composed of mesoporous dendritic silica nanotransporters (DMSNs) loaded with plasmonic NPs composed of AuNPs covered with silver (Ag). This nanocomposite was treated with 4-MPBA for its identification by SERS and with ConA to allow for its binding with bacteria. The “plasmon enrichment module” was developed with Fe_3_O_4_ MagNPs covered with gold (Au) and anti-*S. aureus* antibodies (Fe_3_O_4_@ Au-Ab). This assay was tested on blood samples from mice previously inoculated with *S. aureus*, samples that were incubated at 37 °C with the “enrichment module” for 30 min and then with the “signal module” for 20 min. Captured bacteria were magnetically separated and analysed by SERS, achieving an LOD of 7 CFU mL^−1^ in less than 1 h [145].

### 5.3. MNP-Based Biosensors for the Identification of K. pneumoniae

In 2018, Niu L. et al. developed an LFB based on AuNPs to visualize the presence of specific amplicons of *K. pneumoniae* obtained by isothermal amplification (65 °C) with five pairs of primers designed for the *rcsA* gene, which is specific to this bacterium, through a multiple cross-displacement amplification assay (MCDA). They used a pair of primers, one of which was labelled with fluorescein isothiocyanate (FITC), while the other was labelled with biotin. The researchers immobilized AuNP complexes in the section following the sample loading site, while they fixed an anti-FITC antibody and biotinylated foetal bovine serum albumin (Biotin-BSA) in two test lines. Then, an aliquot of the MCDA test product was loaded in 100 sputum samples (previously analysed by culture and biochemical tests), followed by capillary movement on the NC membrane and the capture of this microorganism (in samples positive for *K. pneumoniae*) in the anti-FITC test line through the interaction between SA-AuNPs and the biotinylated amplicon and biotin-streptavidin affinity. The quality of the device was evaluated by capturing free SA-AuNPs by the biotin-BSA section, achieving an LOD of 100 fg for the MCDA product in <40 min [146].

Recently, a team led by Deb A. (2023) used a specific aptamer to immobilize *K. pneumoniae* (KPBA1) to AuNPs (KPBA1-AuNPs), which demonstrated its usefulness in clinical urine samples (600 µL). Afterwards, 2 mL of KPBA1-AuNP solution was added, followed by incubation for 10 min at room temperature; by Raman spectroscopy, the LOD was 3.4 × 10^3^ CFU·mL^−1^ in 5 min [147].

### 5.4. MNP-Based Biosensors for the Early Detection of A. baumannii

In 2010, researchers led by Yeh CH developed an electromicrochip based on the immobilization of DNA probes specific for different bacterial species (including *A. baumannii*) on slides, followed by the addition of PCR products previously amplified with biotin-labelled primers. Thus, the biotinylated amplicons were recognized by the probes through complementarity, increasing the impedance of the electromicrochip, which was reduced by the addition of AuNPs coupled to streptavidin (increasing their conductance) and significantly increased by the addition of a solution of silver ions. The presence of AuNPs catalysed the precipitation of silver particles, favouring the conductance of the sensor, and changes that were detected with a commercial inductance, capacitance, and resistance (LCR) reader indicated an LOD of 0.825 ng·mL^−1^ (1.2 fM) in ~15 min. The authors noted that this methodology could be applied to other bacterial species [148].

Later, Miller S. et al. (2016) coupled AuNPs with colistin using polyethylene glycol (PEG) as a link between the two components (Col-PEG-AuNPs) and preserved and protected the molecular characteristics of the antibiotic. The interaction between colistin and the outer membrane of Gram-negative bacteria (including *A. baumannii*) is directed primarily towards lipid A, which is the innermost and most conserved constituent of the LPS structure. The authors demonstrated, by electron microscopy, the binding of Col-PEG-AuNPs to the surface of different strains of *A. baumannii* (ATCC^®^ 17978™, ATCC^®^ 19606™ and the colistin-resistant variant ATCC^®^ 19606C™), a process that has been shown to occur in ~7 min [149]. The findings demonstrated that nanometric systems could have great utility in clinical approaches to infectious diseases.

In 2019, a group of researchers led by Bai Y. used recombinant proteins derived from fibres of the bacteriophages *Φ* AB2 (TF2) and *Φ* AB6 (TF6) that had previously been shown to be specific for clinical isolates of *A. baumannii* M3237 and 54149, respectively. These investigators immobilized TF2 and TF6 on aluminium-coated Fe_3_O_4_ MagNPs to form TF2-Fe_3_O_4_@Al_2_O_3_ MagNPs and TF6-Fe_3_O_4_@Al_2_O_3_ MagNPs; both MagNPs were able to distinguish and form complexes with their respective target strains of *A. baumannii* (M3237 or 54149) without interacting with *E. coli* or *S. aureus*, allowing for their magnetic separation and identification by MALDI-MS and reaching LODs of ~10^5^ and ~10^4^ cells mL^−1^, respectively, in ~10 min [150].

In 2020, two groups of researchers used different methods for the detection of *A. baumannii*. Yang S. et al. immobilized a specific aptamer for *A. baumannii* modified on the 5′ end with phosphate (p-Ab-Apt) on Fe_3_O_4_ MagNPs covered with organic metal frameworks (MOFs) based on zirconium (Zr-mMOF), constituting a system called Zr-mMOF-p-Ab-Apt that served as an element for pathogen capture. The element for detection consisted of an aptamer directed to LPS modified on the 5′ end with phosphate (p-LPS-Apt), immobilized on another variant of MOFs called UIO-66-NH_2_ (with a high affinity towards phosphate groups) and previously treated with fluorescein, which was absorbed in these structures (F@UIO-66-NH_2_), thus establishing the F@UIO-66-NH_2_-p-LPS-Apt system. Both elements were evaluated in clinical blood samples obtained from healthy patients inoculated with bacteria (*A. baumannii*, *E. coli*, *S. aureus* and *P. aeruginosa*), where Zr-mMOF-p-Ab-Apt allowed for the capture and selective concentration of *A. baumannii*, and the fluorescent signal was detectable at OD_512_ due to treatment of the F@UIO-66-NH_2_-p-LPS-Apt complex with high concentrations of phosphate anions (1 M NA_2_HPO_4_), which induced the destruction of the UIO-66-NH_2_ nanostructure, allowing for an LOD of 10 CFU·mL^−1^ in ~2.5 h [151].

Farouk F. et al. (2020), synthesized MagNPs followed by surface modification with oleic acid (OA), giving them hydrophobic behaviour with a strong affinity towards bacterial cells. Under this principle, the authors used OA-MagNPs in the culture broths of 93 strawberry samples, which were initially examined by microscopy and Gram staining, followed by the extraction of genomic DNA (gDNA) and PCR amplification of the specific *recA* region of this species. The results were confirmed by sequencing, which identified a total of 14 samples contaminated with *A. baumannii*. In addition, researchers have demonstrated that it is possible to use specific primers for other bacterial species to identify these microorganisms [152].

### 5.5. MNP-Based Biosensors for the Early Identification of P. aeruginosa

In 2017, Žukovskaja O. et al. used AgNPs for the detection of pyocyanin (PYO), a metabolite specifically produced by *P. aeruginosa* that can be found at concentrations of 16.5 µg·mL^−1^ in patients with cystic fibrosis (CF) with lung infections caused by this bacillus [153,154]. They used a microfluidic chip and SERS to analyse water and saliva samples from three volunteers inoculated with different concentrations of PYO, an analyte that aggregated with AgNPs, with an LOD in aqueous solution < 0.5 µM and below 10 µM for in two saliva samples and below 25 µM in one saliva sample [153].

Later (2019), under the same methodological strategy (SERS) and the same detection principle (NP aggregation), but with a matrix of silicon nanowires (SiNWs) basally modified with AgNPs and surface modified with bimetallic NPs (BMNPs; Ag/Au), Žukovskaja O. et al. identified different concentrations of PYO inoculated in artificial sputum medium, finding LODs below 6.25 µM [154].

In the same year (2019), Cernat, A. et al. modified electrodes with a 3:1 ratio of 0.5% agar mixture containing Au/Ag BMNPs. Because of the electroactive properties of PYO, *P. aeruginosa* was detected by electrochemical impedance spectroscopy due to the electrochemical oxidation of PYO caused by BMNPs, which demonstrated the ability to identify the analyte inoculated in commercial samples of serum, whole blood, artificial saliva, and tears, with an LOD of 0.04 μM in 5–10 min [155].

Atta S. and Vo-Dinh T. (2023) used the concept of “mix and detect” from the synthesis of surfactant-free gold nanostars (AuNSs), which were covered with polyvinylpyrrolidone (PVP) and exhibited the ability to absorb, by electrostatic interactions, PYO molecules. Under this principle, the researchers used a solution of these nanostructures (PVP-capped AuNSs) in drinking water, saliva, and urine samples inoculated with PYO, an analyte that can be detected with a portable Raman instrument in 1–2 min, with LODs of 0.05 nM in drinking water and 0.4 nM in urine and saliva samples [156].

### 5.6. MNP-Based Biosensors for the Early Identification of Enterobacter Species

In 2015, Li Y. et al. used the *E. coli* attaching and effacing (*eaeA*) gene as the target gene for the development of an electrochemical biosensor based on DNA for the detection of *E. coli* O157:H7. The authors immobilised DNA sequences with the ability to capture target DNA, and DNA G-quadruplex structures on nanocomposites denominated GOx-Thi-Au@SiO_2_ immobilised on glassy carbon electrodes. The GOx-Thi-Au@SiO_2_ nanocomposites consisted of SiO_2_-coated AuNPs (AuSiO_2_) that were immobilised on graphene oxide (GOx) molecules mediated by thionine (Thi).

Finally, hemin molecules were intercalated into the DNA G-quadruplex structure to allow chemical catalysis peroxidase-like in the presence of hydrogen peroxide. Thus, this system allowed LODs of 0.02–50.0 nM in less than 2 h [157].

For the same bacterial species, Chen Zhou et al. (2018) developed a fibre-optic surface plasmon resonance biosensor using the antimicrobial peptide Magainin I as a specific recognition and capture element for *E. coli* O157:H7. Briefly, the authors used AgNPs conjugated with reduction graphene oxide (AgNPs-rGO), which had previously demonstrated a high SPR response. The AgNPs-rGO nanocomposite was fixed on the surface of the optical fibre after being coated with AuNPs and finally covered with Magainin I.

This fibre-optic biosensor allowed LODs of 5 × 10^2^ CFU·mL^−1^ when used with a Y-type optical fibre patch cord and a spectrometer [158].

Later, Ye Feng et al. developed a multichannel series piezoelectric quartz crystal (MSPQC) sensor for the detection of *E. coli* using a specific region of 16S rRNA as a biomarker. The authors modified gold electrodes consisting of two independent regions; in one of them, they immobilized the capture probe sequence, while, in the other region it was immobilised with a hook probe sequence. Thus, the capture probe recognised the biomarker RNA by complementarity; next, the padlock probe recognised a specific region of 16S rRNA which, in the presence of phi29 polymerase, lead to the formation of long single-strand RNA-DNA products (RCPs).

After, an AuNP-coupled-detection probe was assembled along RCPs, forming a conductive bridge between the two regions of the electrode that, by adding AgNO_3_, led to the significant electrical parameters allowing for LODs of 2 CFU·mL^−1^ [159].

In the same year (2019), Xiao-Zhou M. et al. reported an *E. coli* identification method based on the capacity of this pathogen to capture and reduce exogenous Cu^2+^ to Cu^+^. The produced Cu+ triggered a reaction between azide-modified AuNPs and alkaline-modified AuNPs, and such colour changes allowed for LODs of 10^2^ to 10^7^ CFU·mL^−1^, values that improved with magnetic separation and mass spectrometry (10 CFU·mL^−1^ in 20 min), and that, by integrating them into a smartphone application, allowed for LODs of 40 CFU·mL^−1^ in 1 h [160].

In 2022, M.S. Bacchu et al. immobilised an amine labelled *S. typhi* specific single-strand capture probe on the surface of AuNP. These AuNPs modified were self-assembled on a poly cysteine (P-Cys)-modified screen-printed electrode. The detection process was based on the capture of a target DNA sequence by a probe capture; once the target was captured, the reported probe joined the complex, allowing for LODs of 1 CFU·mL^−1^ in samples of human blood, raw milk, egg, and poultry faeces inoculated with *S. typhi* [161].

## 6. Conclusions

The estimate of 4.95 million global deaths associated with AMR bacteria published in 2019 reflects the adverse global situation that we face as humanity, where the development of new antimicrobial agents against different species of microorganisms has proven to be insufficient in slowing the accelerated growth of this AMR “crisis”, illustrating the urgency of having rapid and accurate diagnostic tools that allow for the early identification of the causative agents of infectious diseases. Such tools could result in timely antibiotic therapies coupled with greater therapeutic success.

The next list of priority pathogens issued by the WHO will promote not only the development of new antimicrobial agents but also the creation of tools, equipment, platforms, or devices focused on the early diagnosis of microorganisms with a high capacity to develop AMR, such as the bacteria included in the ESKAPE group.

Different properties of MNPs, such as their optical, plasmonic, magnetic, electronic, and chemical qualities (among others), have attracted attention for their application in nanomedicine for the development of useful devices for the rapid diagnosis of various diseases. However, the antimicrobial properties of these NMs have been increasingly explored for the possible treatment of bacterial infections, as evidenced by ~89.51% more publications focused on therapeutics (until March 2024), in contrast to those concerning early diagnosis, revealing an important research gap, which is increased by focusing exclusively on the ESKAPE pathogens.

In reports of detection systems based on MNPs, promising results have been shown, with some studies demonstrating the ability to identify ESKAPE bacteria inoculated in biological samples in a few minutes at concentrations ranging from 1 CFU mL^−1^ to concentrations that are undetectable by current diagnostic platforms (Table 1). The high detection power observed in previous studies increases the possibility of developing biosensors capable of determining the antimicrobial susceptibility of isolates, potentially translating not only into early antibiotic therapies but also into timely therapies with the appropriate antimicrobial agent.

In addition, the antimicrobial properties of MNPs, mentioned above, as well as their photothermal properties, can be combined to detect ESKAPE bacteria in vivo and in situ in laboratory animal wound models and, in parallel, exert local antibacterial action mediated by irradiation with NIR, making it possible to develop multifunctional microbiological biosensors capable of identifying minute amounts of ESKAPE bacteria in a short period of time, determine pathogen susceptibility to commonly used antibiotics, and exert antimicrobial activity not only in the host but also in specific areas of infection (targeted therapy), which could be crucial in the battle against AMR.

## Figures and Tables

**Table 1:** Reported detection strategies for ESKAPE pathogens based on MNPS.

ESKAPE Pathogens	Based Biosensor	Combination with Existing Equipment	LODs (CFU·mL^−1^)	Detection Time	Dual Activity (D and T)	Evaluation in Clinical Isolates	References
*E. faecium*	―	―	―	―	―	―	―
*S. aureus*	Colorimetric	MALDI-MS	~10^6^	~1 h	NA	✓	[126]
		UV–Vis S	10	40 min	NA	―	[132]
		NIR laser	―	15 min	✓	―	[131]
	Electrochemical	BPECW	10	30 min	NA	―	[130]
		―	50	~100 min	NA	―	[138]
	LF	RS	8	20 min	NA	―	[135]
		NE	1 × 10^6^	―	NA	―	[133]
		RS	3	―	NA	―	[133]
		NIR camera	18	―	NA	―	[133]
		Thermometer and NIR laser	27	―	NA	―	[133]
		―	10^2^	15 min	NA	―	[140]
	Fluorometric	FM and NIR laser	10	~1.5 h	✓	―	[136]
	Plasmonic	RS and NIR laser	<10^2^	―	✓	―	[134]
		RS	10	―	✓	―	[139]
		RS	11	30 min	✓	―	[142]
		RS	1	~40 min	NA	―	[143]
		RS	7	<1 h	NA	―	[145]
	Molecular	RS and PCR	10^4^ DNA copies	~80 min	NA	―	[137]
	Imaging	EM and MRI	<10^4^	―	NA	―	[144]
*K. pneumoniae*	Colorimetric	MALDI-MS	~10^6^	~1 h	NA	―	[126]
	LF LF	NA	>10^4^	15 min	NA	✓	[129]
		Turbidimeter	24	<40 min	NA	✓	[146]
	Plasmonic	RS	3.4 × 10^3^	5 min	NA	✓	[147]
*A. baumannii*	Colorimetric	MALDI-MS	~10^6^	~1 h	NA	✓	[126]
		NA	―	5 min	NA	✓	[128]
	Conductometric	CT	10–10^3^	2 min	NA	―	[127]
		LCR reader	1.2 fM	~15 min	NA	―	[148]
	Spectroscopic	EM and XDS	―	~7 min	NA	―	[149]
		MALDI-MS	≤10^5^	~10 min	NA	―	[150]
	Fluorometric	FS	10	~2.5 h	NA	―	[151]
	Molecular	PCR	―	―	NA	―	[152]
*P. aeruginosa*	Colorimetric	MALDI-MS	~10^6^	~1 h	NA	―	[126]
	Colorimetric	NA	―	5 min	NA	✓	[128]
	Conductometric	CT	10–10^3^	2 min	NA	―	[127]
		BPECW	10	30 min	NA	―	[130]
	Plasmonic	MF and RS	<10 µM	―	NA	✓	[153]
		RS	6.25 µM	―	NA	―	[154]
		RS	<0.4 nM	1–2 min	NA	―	[156]
	Electrochemical	EIS	0.04 μM	5–10 min	NA	―	[155]
Genus *Enterobacter*	Colorimetric	MALDI-MS	~10^6^	~1 h	NA	―	[126]
		NA	―	5 min	NA	✓	[128]
		NIR laser	―	15 min	✓	―	[131]
		UV–Vis S	10	40 min	NA	―	[132]
		MS	10	20 min	NA	―	[160]
		Smarthphone	40	1 h	NA	―	[160]
	Plasmonic	Spectrometer	5 × 10^2^	―	NA	―	[158]
	LF	NA	>10^4^	15 min	NA	✓	[129]
	Electrochemical	CT	10–10^3^	2 min	NA	―	[127]
		EWS	0.02–50 nM	<2 h	NA	―	[157]
		―	2	―	NA	―	[159]
		SECI	1	―	NA	―	[161]

LF = Lateral flow; D and T = Diagnosis and therapeutic; CFU·mL^−1^ = Colony forming units per millilitre; BPECW = Bipotential electro-chemical workstation; LCR = Inductance, capacitance, and resistance; MRI = Magnetic resonance imaging; NE = Naked eye; NA = Not applicable; PDR = Pandrug-resistant; ― = Not specified; CP = Carbapenemase-producing; UV-Vis S = UV–Vis spectrophotometer; RS = Raman spectrophotometer; FM = Fluorescence microscopy; EM = Electron microscopy; XDS = X-ray energy dispersive spectroscopy; MS = Mass spectroscopy; FS = Fluorescence spectrophotometer; CT = Conductometric transducer; MF = Microfluidic platform; EIS = Electrochemical impedance spectroscopy; EWS = Electrochemistry workstation; SECI = Spectro electrochemical instrument.
